# Evolving Diagnostic and Treatment Strategies for Pediatric CNS Tumors: The Impact of Lipid Metabolism

**DOI:** 10.3390/biomedicines11051365

**Published:** 2023-05-05

**Authors:** Paula Fernández-García, Gema Malet-Engra, Manuel Torres, Derek Hanson, Catalina A. Rosselló, Ramón Román, Victoria Lladó, Pablo V. Escribá

**Affiliations:** 1 Laboratory of Molecular Cell Biomedicine, University of the Balearic Islands, 07122 Palma de Mallorca, Spain; paula.fernandez@uib.es (P.F.-G.); gema.malet@uib.eu (G.M.-E.); manuel.torres@uib.es (M.T.); ca.rossello@uib.es (C.A.R.); ramon.roman@uib.es (R.R.);; 2 Laminar Pharmaceuticals, Isaac Newton, 07121 Palma de Mallorca, Spain; 3 Hackensack Meridian Health, 343 Thornall Street, Edison, NJ 08837, USA; derek.hanson@hackensackmeridian.org

**Keywords:** pediatric CNS tumors, pediatric brain tumor and treatment, melitherapy, membrane lipid therapy, molecular basis of pediatric tumor, clinical trials

## Abstract

Pediatric neurological tumors are a heterogeneous group of cancers, many of which carry a poor prognosis and lack a “standard of care” therapy. While they have similar anatomic locations, pediatric neurological tumors harbor specific molecular signatures that distinguish them from adult brain and other neurological cancers. Recent advances through the application of genetics and imaging tools have reshaped the molecular classification and treatment of pediatric neurological tumors, specifically considering the molecular alterations involved. A multidisciplinary effort is ongoing to develop new therapeutic strategies for these tumors, employing innovative and established approaches. Strikingly, there is increasing evidence that lipid metabolism is altered during the development of these types of tumors. Thus, in addition to targeted therapies focusing on classical oncogenes, new treatments are being developed based on a broad spectrum of strategies, ranging from vaccines to viral vectors, and melitherapy. This work reviews the current therapeutic landscape for pediatric brain tumors, considering new emerging treatments and ongoing clinical trials. In addition, the role of lipid metabolism in these neoplasms and its relevance for the development of novel therapies are discussed.

## 1. Introduction

After hematological cancers, pediatric central nervous system (CNS) or neurological tumors, including pediatric brain tumors (PBTs), are the second most common childhood malignancies. The incidence of pediatric neurological cancers is around 3–6 cases per 100,000 children [1,2], one-third of the rate in adults. PBTs are the main cause of death among all childhood cancers [3,4]. Gliomas account for half of PBTs, followed by neuronal tumors (33%) and embryonal tumors (15%). The molecular basis of PBTs may differ considerably from those in adults, although they share common characteristics [5,6]. The updates of the WHO classification of pediatric CNS tumors in 2016 incorporated histological data and information regarding the molecular signature of specific tumor subtypes. The last update in 2021 [5] included the findings in pediatric tumor genomics, showing the rapid transition from a mainly microscopic to a molecular classification [6,7] (Table 1). This helped to define new tumor subgroups and changed the diagnosis and treatment landscape [8,9,10].

Like adult brain tumors, the treatment for pediatric brain and other CNS tumors includes radiotherapy, surgery, and chemotherapy [34]. The challenge posed by pediatric neurological tumors in the context of basic and clinical research accounts for the multiple clinical trials developing chemotherapies that still rely on cytotoxic agents, but also new drugs in development encompass innovative and multidisciplinary approaches, from cancer vaccines to lipid-based therapies. Despite the hurdles that limit the progress of drug-development for PBTs, rapid regulatory changes and close international cooperation are expected to favor PBT research and improve the outlook for patients [35,36]. Currently, improvements in the diagnosis and treatment of PBTs have led to a cure rate above 50% [37], which may be increased with new therapeutic approaches.

Alterations to lipid metabolism are a hallmark of cancer and of brain tumors in particular, whereby lipogenesis, lipid uptake, and lipid storage are upregulated [38,39,40,41,42,43,44]. However, we are only now beginning to understand how this may affect neurological tumor progression in children. In addition, the plasma membrane composition of brain cancer cells differs from that of healthy cells, which has a direct impact on proliferative signal transduction [44,45,46,47,48]. Targeting pathways that regulate lipid metabolism provides a novel strategy to combat PBTs, which will require a better understanding of lipid metabolism.

As such, this review provides an overview of the potential relevance of lipids in the diagnosis, classification and treatment of PBTs. We will summarize the recent advances in the genetics associated with PBTs and how they could be related to lipids, the weight of lipid metabolism in prognosis, the new advances in diagnostic and imaging tools based on lipid content and the endeavors to develop new treatments for PBTs that have entered clinical trials. In this context, we provide a perspective on how understanding the dysregulation of lipid metabolism associated with PBTs may shed light on future diagnostic and therapeutic outcomes.

## 2. Cytogenetic Alterations and Lipidomic Landscape of Pediatric Neurological Tumors 

The classical nomenclature for classifying gliomas as low-grade (LGG) or high-grade (HGG) has been applied in both adults and children. However, in the last decade, it has become increasingly evident that there are key genetic differences between adult (LGGs/HGGs) and pediatric (pLGGs/pHGGs) gliomas in terms of onset, location, clinical outcome and histopathological features [49,50]. Recent advances in analytical techniques enable tumor genome sequencing and methylation profile, paving the way towards precise diagnosis and personalized therapies. The relevance of these molecular characteristics has led to a new classification based on them rather than on histopathological characteristics [6,51], sorting PBTs beyond their designation as pHGG or pLGG. Indeed, the suitability of including the molecular features of the tumors, as shown in Table 1, is seen in the fact that many of the genes involved in the genetic alterations of CNS tumors encode for proteins that are somehow regulated by lipids or, conversely, that modulate lipid metabolism or composition (Table 1, e.g., Dicer1, CD24, SWI/SNF, P53 or Cyclin D1) [11,52].

Although progress has been made in the identification of PBT driver genes and those with diagnostic value, many targets remain to be discovered, in particular genes related to disease progression, response to treatment, metastasis and relapse [53]. While this classification focuses on the genomic perspective, many of the genetic or proteomic alterations could have an impact on the lipidomic landscape.

Pediatric cancer genomes are characterized by a heterogeneous group of genetic alterations that include germline and somatic mutations, gene fusions, deletions, abnormal gene expression, chromosomal rearrangements, and altered methylation patterns (Table 1) [34,54,55,56,57]. The histone H3 mutation status of the tumors has led to the new classification of the different types of tumors in the pediatric-type diffuse high-grade glioma family, considering the associated genetic alterations and modifications to cell signaling (Table 1) [6,58]. There are two main variants of histone H3 identified in pediatric-type diffuse high-grade gliomas besides the H3 wild-type: the K27 alteration (inducing H3.3K27M expression and promoting genomic instability [59]) and the G34V mutation (inducing H3.3G34V expression and in turn affecting methylation at K36 and K27, and inducing gliomagenesis with a worse prognosis [60,61]). Apart from the characterization of the mutation/methylation status of histone H3 and a low proportion of pediatric-type diffuse high-grade gliomas carrying IDH mutations, there are also a series of genes defining the tumor molecular signature that can be altered as a consequence of a constitutional deficiency for DNA repair which may (1) serve as regulators of the lipid metabolism and participate of the change in the tumor lipid composition, such as TP53 [11,12], (2) act as transmembrane proteins whose location and activity are affected by membrane lipid structure and composition, such as NTRK, or (3) be regulated by lipid modifications and enzymes involved in lipid metabolism, e.g., EGFR and MCYN [13,15]. In view of this, the development of potential anti-tumor treatments aimed at modulating lipid metabolism and/or membrane composition is not surprising. On top of that, the retention of a wild-type IDH genotype supports lipid biosynthesis and preserves the antioxidant level’s rise during the tumor growth [14].

Pediatric-type diffuse low-grade glioma family tumors (formerly pLGGs) are essentially as common as malignant gliomas and embryonal tumors but are expected to have a better prognosis [62]. Considering that, even if the 10-year median survival is higher than 90%, the challenges regarding their treatment consist of avoiding recurrences and long-term sequelae after surgery, radio- and chemotherapy. This family includes different tumor types according to their molecular characteristics with different outcomes and signaling pathways altered, non-related to IDH mutations as opposed to their adult counterparts, and frequently associated with an enhanced MAPK pathway and BRAF gene alterations [6,63].

Interestingly, BRAF regulation is closely related to the lipogenic pathway, polyunsaturated free fatty acid (PUFA) metabolism, and lipid droplet accumulation in the cells [16,17], while the MAPK pathway is tightly controlled by lipid membrane modifications that regulate the activity of growth factor receptors as well as signaling intermediates (such as Ras) [18]. Another family of pediatric gliomas also characterized by BRAF mutations and MAPK enhancement is the circumscribed astrocytic gliomas, which include pilocytic astrocytoma (PA), frequently associated with KIAA1549:BRAF gene fusion [64], and pleomorphic xanthoastrocytoma (PAX), commonly carrying BRAF mutations and homozygous CDKN2A deletion [65].

Moreover, mutations in the fibroblast growth factor receptor (FGFR) family have been identified and characterized in different pediatric brain tumor types via large-scale genetic analysis, such as recurrent FGFR1 somatic mutations (N546K and K656E), FGFR1–TACC1 gene fusions, and duplications of the FGFR1 tyrosine kinase domain in patients with PA and dysembryoplastic neuroepithelial tumors (DNETs) [66]. In addition, their membrane location has been associated with malignancy and tumor grade [66]. Finally, these receptors activate a lipid-anchored Grb2-binding protein (FRS2) that ultimately triggers MAPK pathways as well [67].

In addition, there are other signaling pathways than the MAPK axis (activated by BRAF and FGFR mutations, among others) which are altered in the pediatric-type diffuse low-grade gliomas—for example, MYB/MYBL1 amplifications and rearrangements have been identified in diffuse astrocytomas, becoming a new type of tumor in the last WHO classification update.

On the other hand, unlike most adult gliomas, a notable fraction of PBTs have a hereditary component. For example, subependymal giant cell astrocytomas (SEGAs) are closely associated with germline mutations in the TSC1/TSC2 genes, whose defective phenotype could be reversed by inhibiting lipophagy, as has been shown in mouse models, where this inhibition reduces tumorigenesis [20]. In addition, in the ependymal tumor family, the majority of the tumors belong to the supratentorial ependymomas ZFTA fusion-positive containing a C11ORF95-RELA gene fusion, while the remainder harbor fusions involving the YAP1 oncogene (supratentorial ependymomas, YAP1 fusion-positive) [68]. RELA encodes the p65 subunit of the nuclear factor-κB (NF-κB), one regulator of the inflammatory response that can be activated by saturated fatty acids and lipid peroxidation-derived aldehydes [69,70]. On the other hand, YAP1 plays an important role in the lipid metabolism adaptation during tumorigenesis, acting in different pathways such as its activation by stearoyl-CoA-desaturase-1 (SCD1) and its interaction with beta-catenin, among others [71].

Finally, embryonal tumors are a heterogeneous group of malignant neuroepithelial tumors making up about 15–20% of the pediatric CNS tumors (WHO grade 4). Embryonal tumors include medulloblastoma, atypical teratoid rhaboid tumors (ATRTs) and embryonal tumors with multi-layered rosettes (ETMRs) (Table 1, [72]). Medulloblastoma is the most frequent embryonal tumor and the second most common PBT, with frequent MYC amplifications [55]. Fatty acids act as inhibitors for the DNA-binding c-Myc/Mas dimer, being a potential treatment for this type of PBT [28]. The latest classification divided medulloblastoma into four genetically defined subsets based on the oncogenic profile of the CTNNB1/APC (WNT activated), SMO/GLI2 (SHH activated) with or without and TP53 mutation signaling pathways. All of these pathways are modulated by different lipid species and their metabolism or, conversely, as happens with beta-catenin and p53, can control the lipid catabolism (Table 1).

## 3. Lipid Metabolism and Pediatric Brain Tumor Prognosis

Lipid metabolic reprogramming is an established hallmark of cancer progression and recent findings suggest that it may influence therapeutical response and resistance [73]. In addition to the above-mentioned relationship between the alterations implicated in CNS tumors and the lipid metabolism or lipid composition, other regulation pathways must be considered. Microdomain localization and activity of transmembrane proteins are likely to be affected by lipid membrane composition (Ntrk), as are those proteins that are regulated by lipidation (EGFR, K-Ras), since their lipid modifications show preference for different types of membrane microdomains, which in turn depend on membrane lipid composition [13]. Altered lipid metabolism or content may affect the general activity of cells, influencing several proteins involved in the pathologic profile of CNS tumors. Alterations to the β-oxidation pathway (e.g., β-catenin malfunction) are implicated in general cell metabolism. For example, those affecting the production of acetyl-CoA are involved in the Krebs cycle, fatty acid metabolism, acetylcholine synthesis, acetylation, etc. Accordingly, the regulation of lipid metabolism or membrane lipid composition offers therapeutic advantages with respect to the possible emergence of resistance and possibly greater guarantees of success. Indeed, the clear relationship between the genetic alterations described in pediatric patients with CNS tumors and the alterations in lipid metabolism highlights the importance of lipids as potential targets for new therapies to manage these conditions.

The role of lipids in cancer cell signaling has been highlighted recently and alterations to the expression of genes that influence lipids are at least as relevant to tumorigenesis as those in conventional oncogenes [74].

In oncological research, gene expression datasets have provided relevant information that helps us to understand the molecular basis of different cancers, identify new therapeutic targets and biomarkers, as well as define prognosis [42,75]. However, there are very few PBT datasets available, which limits the potential advances in this field.

**Figure 1:**
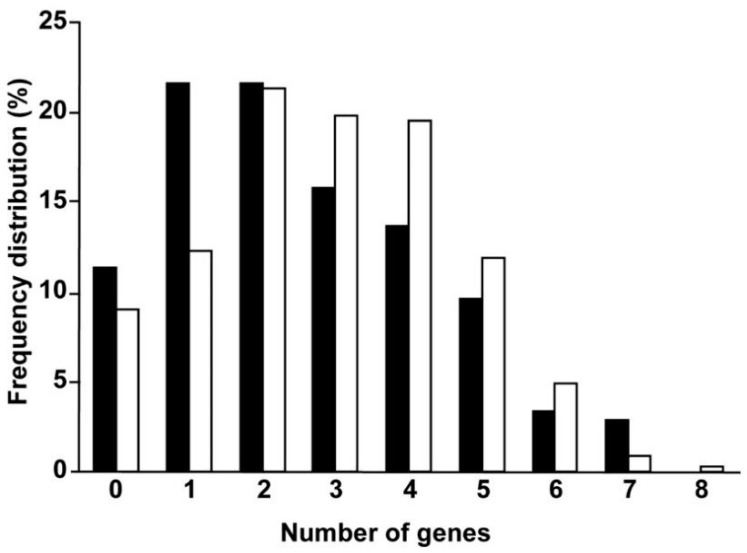
Frequency distribution of alterations to gene expression in glioma patients. Eleven lipid metabolism genes (ASAH2, FASN, SMPD2, SMPD3, PHYH, SPTLC3, SGMS1, SGMS2, SPHK1, SPHK2, UGCG) and eleven conventional oncogenes (AKT1, AKT2, AKT3, MYC, MAPK13, PIK3CB, PI3KCG, PI3KCD, NRAS, KRAS, TNF) with significantly altered expression were analyzed. The frequency of the alterations in expression (either higher or lower) was analyzed in patients with glioma in the REMBRANDT database. The graph shows data from 0 to 8 alterations in lipid metabolism (closed bars) or conventional oncogene (open bars) transcripts. Reprinted/adapted with permission from Ref. [74] Copyright 2014, Elsevier.

In a study of adult gliomas [74], the expression of eleven genes involved in lipid metabolism (e.g., SGMS, FASN, SPHK, etc.) was significantly altered and their profiles were compared to those of eleven oncogenes that were also significantly affected (e.g., AKT, MYC, RAS, etc.). In these patients from the REMBRANDT database, the probability of developing glioma when the expression of one gene that affects lipids is altered was approximately 21%, whereas the probability of developing glioma with alterations to one conventional oncogene was approximately 12% (Figure 1), suggesting that the genes involved in lipid metabolism may have a strong impact on tumorigenesis. Moreover, the median survival time of patients was more strongly affected by alterations to lipid-related genes rather than conventional oncogenes. These results suggest that the altered expression of genes involved in lipid metabolism could have a similar or stronger influence on the development of brain tumors than oncogenes.

Lipids are major structural elements in the brain, with fatty acids making up about 50% of the total mass of CNS membranes. Deregulation of fatty acid uptake and lipid metabolism has been described in malignant adult gliomas, resulting in marked differences in lipid metabolism between LGGs and HGGs [76]. Membrane lipids define the type and abundance of peripheral proteins that bind to membranes, influencing the propagation of signals driving proliferation or other events [46,77]. Recently, cerebrospinal fluid was explored for potential metabolites and proteome biomarkers of childhood brain tumors, showing that 6% of the proteins altered in the cerebrospinal fluid from extraventricular drainage in pediatric and brain tumor samples, compared to control samples, were associated with membranes [78]. Even more importantly, the metabolic and lipidomic profile of cerebrospinal fluid from patients with medulloblastoma, the most common malignant brain tumor in childhood, are distinctive in terms of their TNF-beta, TNF-alpha, and adipogenesis signatures, allowing the classification of patients with medulloblastoma by analyzing their cerebrospinal fluid and comparing it to that of normal patients [79].

**Figure 2:**
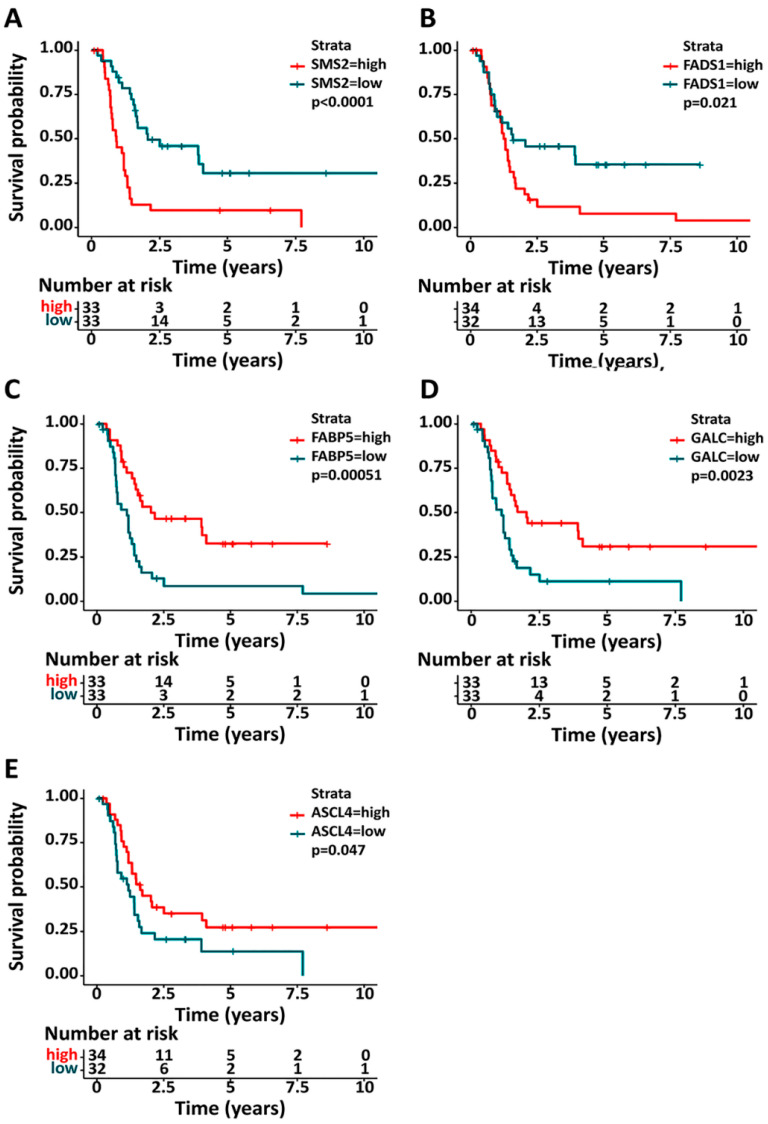
Overall survival and gene expression in pediatric gliomas. Kaplan–Meier plots showing the OS in pediatric patients with gliomas with high (red) or low (green) expression of SMS2 (**A**), FADS1 (**B**), FABP5 (**C**), GALC (**D**) and ASCL4 (**E**). Survival probability was considered significantly different at *p* < 0.05 (log rank test). See text for further details.

To determine the impact of genes involved in lipid metabolism on the survival of pediatric glioma patients, we analyzed the expression of genes controlling membrane lipids. The raw dataset on the XENA website was used (http://xena.ucsc.edu/ (accessed on 8 September 2019)), available at the Array Express data repository of the European Bioinformatics Institute (http://www.ebi.ac.uk/arrayexpress/ (accessed on 8 September 2019)) under the accession number E-TABM-1107 [80]. RNA and clinical data were considered to analyze the genes related to lipid metabolism, and only those patients with a known overall survival (OS) were included. A total of 66 pediatric patients were studied, of whom 17 were censored for the Kaplan–Meier determination, generating Kaplan–Meier curves using the “survival” and “survminer” packages in the R software. Not all of the selected genes associated with lipid metabolism were included in the array (Table 2). For each of the genes selected, patients were divided into “Low” or “High” groups to reflect expressions above or below the median expression of the whole population. Kaplan–Meier curves were produced for all of these genes and significant differences in median OS were evident for five genes related to lipid metabolism based on their expression when the log-rank test was used (SMS2, FADS1, FABP5, GALC and ACSL4; *p*-value < 0.05: Figure 2). 

The SMS2 gene encodes sphingomyelin synthase 2, one of the two isoforms (SMS1 and SMS2) which mediate sphingomyelin (SM) production [81]. SMS2 expression was previously related to the survival of adult glioma patients, where lower expression is associated with a better prognosis [82]. Interestingly, similar results were obtained in pediatric patients (Figure 2A), although unfortunately, the databases used did not contain data on SMS1, which is associated with better prognosis in adult patients. Nevertheless, this represents further evidence that membrane sphingolipids are critical for brain tumor cell proliferation.

FADS1 (Fatty Acid Desaturase 1) enzyme regulates fatty acid unsaturation and controls the metabolism of inflammatory lipids such as prostaglandin E2. Interestingly, low FADS1 expression in pediatric glioma patients correlates with better prognosis, with a higher 2- and 5-year survival, yet these differences were only evident in patients that survived at least 2 years (Figure 2B). Inhibiting FADS impairs cancer cell proliferation, and it has been proposed as a potential antitumor strategy [83,84]. 

The FABP5 gene encodes a fatty-acid-binding protein that preferentially transports saturated fatty acids and retinoic acid to the cytoplasm and nucleus [85]. FABP5 is expressed in the mid-term embryonic rat brain, peaking at birth before gradually declining in post-natal life. Contrary to FABP5’s role as tumor promoter in some types of cancer, such as cervical cancer or hepatocellular carcinoma [74], and as a malignancy element in adult LGG via NF-κB pathway activation [86], in the pHGG dataset studied, higher FABP5 expression was associated with a better prognosis (Figure 2C), pointing out the specific molecular pattern followed by pediatric CNS tumors.

The GALC gene encodes a lysosomal galactose-ester-bond hydrolase for galactosylceramide, galactosylsphingosine, lactosylceramide and monogalactosyldiglyceride, and constitutes a marker for mature cells of oligodendroglia [87]. Although this protein has been described as a pro-angiogenic factor that may negatively influence cancer progression [88], a positive association was evident between high GALC expression and median survival in pediatric glioma patients (Figure 2D). 

The protein encoded by the ACSL4 gene is an isozyme of the long-chain fatty-acid-coenzyme A ligase family converting free long-chain fatty acids into fatty acyl-CoA esters in what is a key reaction in lipid biosynthesis, fatty acid metabolism and fatty acid uptake. Similarly to what has been described in adult patients, high expression of this gene in pediatric patients is associated with a better prognosis [89]. In particular, high ACSL4 expression is associated with a significantly higher 5-year survival (Figure 2E).

**Figure 3:**
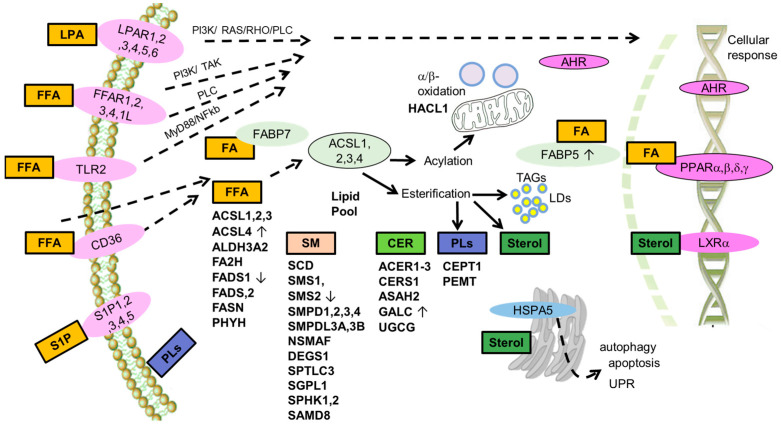
Metabolic pathways regulating lipid metabolism in PBTs, and specific molecular signatures related to plasma membrane receptors, nuclear receptors, and the metabolism of specific lipid subtypes. Arrows indicate good (↑) or bad (↓) prognosis associated with the high expression of ACSL4, GALC, FABP5, FADS1, and SMS2. CER: ceramide FFA: free fatty acids, LDs: lipid droplets, LPA: lysophosphatidic acid, PI3K: phosphatidyl inositol kinases, PLs: phospholipids, PLC: phospholipase C, SM sphingomyelin, S1P: sphingosine phosphate, UPR: unfolded protein response.

Both SMS2 and GALC are involved in sphingolipid metabolism, while FABP5, FADS1 and ACSL4 are involved in fatty acid biosynthesis, metabolism and transport. Targeting sphingolipids has been proposed as a treatment for GBM patients, including pediatric ones [90]. For sphingolipids, low SMS2 expression and high GALC expression are associated with a better prognosis, and both of these conditions are related to an increase in ceramides [91]. In the case of fatty acids, the better prognostic signature of high ACSL4 and FABP5 and low FADS1 (Figure 2E) might be explained by the increased cellular uptake of arachidonic acid at high ACSL4 expression [92] and the increased pool of saturated fatty acids upon high FABP5 expression and low FADS1 expression [93]. Saturated fatty acids represent a more efficient cellular fuel than unsaturated fatty acids, and the higher energetic demands of cancer cells may partially justify this inverse association with pediatric patient survival. Deregulated fatty acid synthesis may contribute to cancer in many ways, modifying membrane lipid composition or the provision of substrates for ATP production, influencing signaling pathways involved in inflammation and in cell differentiation, proliferation, and survival [92]. Thus, some of the genes involved in lipid metabolism studied here showed similar trends in pediatric and adult brain tumors, while FABP5 and GALC expression was unexpectedly related to OS. Hence, certain molecular features of PBTs might differ from those in adult patients, and further studies will be necessary to reveal the full role of genes involved in lipid metabolism in cancer cells. It is not surprising that molecular differences occur between pediatric and adult brain tumors given the increase in genetic aberrations with age. Therefore, a lower mutation frequency and stronger requirement for lipogenesis in the developing brain is expected in children with cancer. Some of these genetic differences between pediatric and adult brain tumors were highlighted above and they anticipate that significantly different treatments may be necessary for PBTs with respect to their adult counterparts. Indeed, it is noteworthy that such a small patient population (only 66 patients evaluated) could display significant differences in the expression of certain genes that might be useful for diagnosis. Additionally, databases containing information from more patients could reveal more genes involved in lipid metabolism that might be associated with survival. The context within which the prognostic genes are integrated in the lipid metabolism landscape is schematically illustrated in Figure 3.

In light of the results that show the relevance of the expression levels of genes related to lipid metabolism in the prognosis of patient survival, it is logical to suggest that another control point of special interest is the transcription factors that regulate the expression of these genes. Examples of transcription factors that regulate the expression of mediators of lipid metabolism are listed here as potential biomarkers of PBT prognosis and survival (Table 3).

## 4. Neuroimaging of Lipids in Pediatric Brain Tumors

As lipids are a major component of brain tissue, the lipid profile of a cell is a molecular signature or its cell type, and its growth and differentiation status. The lipid composition of brain tumors differs from that of healthy brain tissues and reflects the lipid metabolism reprogramming of cancer cells [115,116,117,118]. Transformed cells have higher lipid uptake and storage (often stored in lipid droplets), increased levels of choline-containing compounds, higher lipid biosynthesis and a lipid-dependent catabolism [44]. The rewiring of lipid metabolism through cancer progress has been widely studied in adults and to a lesser extent in children. Nevertheless, the battery of neuroimaging techniques for the analysis of PBTs has been increasing exponentially in recent years, providing powerful non-invasive tools to monitor the biochemical composition of tumors and providing valuable information for diagnosis, surgery planning and for post-surgery follow-up. The analysis of the protein and lipid profile of tumors is able to (1) distinguish between healthy and tumor tissue, defining the tumor contour, (2) identify and diagnose specific tumor types and sub-type, and (3) monitor changes in the biochemical composition of tumors associated with tumor progression and response to treatments.

Raman spectroscopy has been applied to assess the alterations in the protein and lipid content in tumors, as well as defining their morphology, discriminating between medulloblastoma and healthy tissues [119]. Additionally, this technique has proven useful in discriminating medulloblastoma from several types of low- and high-grade tumors such as astrocytoma, glioma, ganglioglioma and medulloblastoma [120]. For this reason, the metabolic profiles of different types of lipid peaks (sphingomyelin, phospholipids, cholesterol, etc.) are a valuable tool to distinguish healthy brain tissue from malignant tissue as well as to distinguish among different types of tumors.

In this regard, in vivo magnetic resonance spectroscopy (MRS) detects lipid peaks that resonate at 1.3 ppm and 0.9 chemical shift in 1H MR spectra. These peaks correspond to the methylene and methyl signals from CH2 and CH3 groups, respectively, in fatty acyl chains of triacylglycerides. Thus, the lipid peaks indicate tissue damage (necrosis) and release of membrane lipids and of cytoplasmic mobile lipids contained in lipid droplets of intact cancer cells. Instead, the choline-containing compounds present in the membranes do not correspond to those peaks but to molecules participating in phospholipid metabolism [121,122]. Magnetic resonance imaging (MRI) helps to predict the tumor type and grade, while high-resolution proton magnetic angle spinning spectroscopy (HRMAS) has been applied to the study of PBT biopsies. Recent studies using HRMAS have examined the metabolic profile of ependymoma, medulloblastoma and pilocytic astrocytoma samples, demonstrating a correlation between choline-containing lipid compounds with tumor grade (glycerophosphocholine, phosphocholine, choline) [123], supporting the results found in different PBTs using the total choline levels determined by MR spectroscopy as a diagnosis tool for neuropathologies in children [124,125]. Choline is required for phospholipid synthesis and is a marker of cell membrane integrity and density, and increased choline levels are thought to reflect increased cell turnover [126]. The levels of phosphocholine may be associated with changes in membrane composition and structure, which may in turn have an impact on cell proliferation. Indeed, medulloblastoma (grade 4) had higher levels of choline-containing compounds than pilocytic astrocytoma (grade 2) and ependymoma (grade 1), while there were fewer fatty acids as the tumor grade worsened.

Lipidic profiles of tumors obtained by MSI (magnetic spectroscopy imaging) have been used to distinguish medulloblastoma and pineoblastoma, with glycerophosphoglycerols and sphingolipids considered the best markers, respectively [127]. Several studies have shown phosphatidylcholine levels to be significantly higher in medulloblastoma and glioblastoma, and in LGG, than in tissue from healthy individuals. Proton NMR analysis demonstrated that the total cholesterol and choline-containing phospholipid levels of medulloblastoma patients (and other adult brain tumor types) were higher than those in blood serum and tumor tissue from healthy individuals [128].

**Figure 4:**
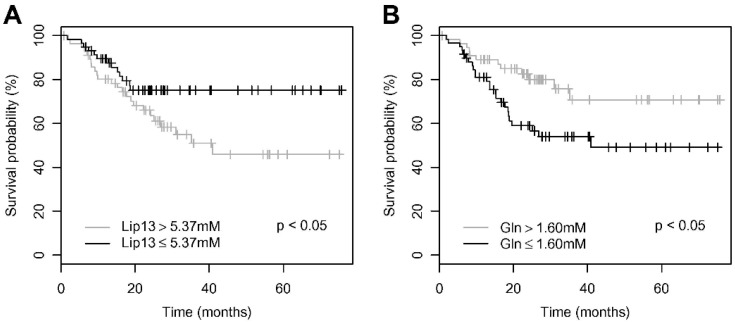
Survival of PBT patients in relation to the metabolite profiles measured by magnetic resonance spectroscopy in vivo for a range of tumor types. Kaplan–Meier survival plots for (**A**) lipids at 1.3 PPM, (**B**) glutamine of 115 pediatric patients study followed up for a median of 35 months. Significance values assessed using a chi squared test for equality. Reproduced with permission of Martin Wilson [129].

The tumor lipid levels measured by MR spectroscopy are strongly associated with tumor grade, but importantly, they also predict the survival of children with brain tumors [129]. In the study reported by Wilson et al., in a cohort of 115 pediatric patients diagnosed with different types of PBTs (gliomas and embryonic tumors), lipid levels were correlated with survival but also with the glutamine content in the tumor. This correlation suggests a possible link between tumor development and lipogenesis where glutamine serves as a carbon source (Figure 4).

The levels of lipids alone or in combination with choline-containing compounds (signals from choline, phosphocholine and glycerophosphocholine) were also explored using proton magnetic resonance spectroscopic imaging (MRSI) in 76 children with PBTs of diverse types of malignancies, indicating possible alterations to the phospholipid metabolism of tumor cells [130]. In this in vivo analysis, MRSI was used in conjunction with an analysis of tumor grade via standard histopathology. Brain MRSI measurements in vivo show that tumor lipid levels are associated with tumor grade and predict the survival of children with brain tumors.

In another study reported by Bennet et al., HRMAS was used to obtain the ex vivo metabolite profiles of a cohort of 133 pediatric patients diagnosed with a wide variety of different patient brain tumor types (gliomas, ATRT, medulloblastoma among others). In this study, high intratumor lipid levels measured ex vivo by HRMAS correlated with a poor OS in children, confirming the clinical value of lipid metabolite profiling for PBTs as prognostic markers [131].

Moreover, recent data using proton magnetic resonance spectroscopic imaging (MRSI) of child brain tumors in vivo demonstrated a prediction of the outcome of treatments based on the choline-containing compounds, lipids, lactate, and N-acetyl aspartate peaks [132]. The findings of this study showed that patients who responded to chemotherapy or radiation displayed higher total creatine and lower choline, lactate and lipid levels than the patients who did not respond to treatment or were not treated.

Finally, although there is little information as to how lipid metabolites may be differentially associated with PBT metastasis development, recent evidence suggests that lipids are differential components of metastasizing as opposed to non-metastasizing medulloblastomas in mouse models analyzed via 3D-MALDI MS (three-dimensional matrix-assisted laser desorption/ionization mass spectrometry imaging) [133]. This technique allowed the identification of the spatial distribution of lipids within the metastasis sites including phosphatidylserine, phosphoinositides and phosphatidylethanolamines. This and future studies in this line can not only provide relevant information on the mechanistic steps of the metastasis progress of PBTs, but could also define biomarkers and targets that might prove fruitful for the development of novel therapies.

As a result of the abovementioned studies, the application of imaging plus other metabolomic techniques to the field of pediatric oncology is likely to elucidate the lipid metabolic pathways that contribute to the malignant transformation of PBTs, providing complementary information to standard histopathology and genetics that will surely contribute to the classification and treatment of PBTs in the future.

## 5. Current Trends in the Treatment of Pediatric Neurological Tumors Targeting the Lipid Metabolism

The treatment of pediatric neurological tumors requires a multi-disciplinary approach that may incorporate interventions involving neurosurgery, radiotherapy, and chemotherapy [34]. The specific treatment for an individual PBT depends on the type, size, and location of the tumor, as well as the child’s age and overall health. Several advances in neurosurgery have improved the success rates of tumor surgery, including image guidance, functional mapping, neuroendoscopy, and ultrasonic aspiration [134,135]. Surgical excision is the initial approach recommended in all cases except those in which the tumor is close to vital structures or due to the infiltration of the tumor, as is the case of diffuse midline glioma H3K27M-mutant (formerly classified as diffuse intrinsic pontine glioma [DIPGs]) [136]. Depending on the type of the tumor, its location, and the percentage of resection achieved, the pediatric patient can be administered radiotherapy, with all of the secondary effects associated and variable efficacy outcomes [137,138,139,140,141,142].

While surgery and radiotherapy were initially the two mainstays of PBT therapy, chemotherapy has taken on a more important role in the past 30 years, although it still often involves the use of non-specific cytotoxic conventional chemotherapy. However, there are determined chemotherapy drugs preferred for each of the different kinds of PBTs [34], during or after the radiotherapy treatment, based on the side effects induced, the signaling pathway targeted by the treatment, and the prognosis and sensibility to the drug of the tumor, which do not need to be related to the outcomes observed in adult brain tumors [136,141,142,143]. For instance, regarding the treatment of the CNS embryonal tumor family, adjuvant lomustine, vincristine, cisplatin and cyclophosphamide are used to treat medulloblastomas, while intensive treatment with alkylating agents, high-dose methotrexate and high-dose chemotherapy with stem cell rescue are used to treat ATRTs and ETMRs [84]. On the other hand, chemotherapy plays a palliative role in diffuse midline glioma H3K27M-mutant tumors, yet there is no established role for systemic chemotherapy to treat craniopharyngioma [144].

Considering that the best approach to deal with the pediatric neurological tumors is still tumor resection as much as possible, and that prognosis is worse in patients with local residual or disseminated disease compared to patients with no evidence of disease after surgery [145,146], new therapies that can the pass through the BBB [147,148,149] to reach and reduce CNS tumors are under study [150,151]. The tight junctions of the endothelial cells in cerebral capillaries impair the mobilization of large polar compounds between the blood and brain tissue. By contrast, small non-polar lipid-soluble compounds rapidly traverse the BBB and hence, in addition to their safety and efficacy, small hydrophobic drugs used in membrane lipid therapy (melitherapy) have tremendous potential to treat pediatric CNS tumors [46,49].

Currently, several targeted therapies are under clinical trials to treat PBTs and other pediatric neurological tumors, yet since 2010–2011, only the mTORC1 inhibitor everolimus has been approved by the EMA and FDA for the treatment of SEGAs [152]. To date, most of the drugs have been tested as off-label treatments and no drugs have been specifically designed for children with neurological tumors from scratch, although there has been much recent progress in the generation of PBT cell models, patient-derived orthotopic xenografts and biobanks [153,154]. For example, a combination of temozolomide, which is used in the standard of care treatment of glioma in adults, with O6-benzylguanine has been investigated for the treatment of children with refractory or recurrent brain tumors (NCT00052780). A wide variety of other new therapies are currently being tested in clinical trials, including mutation-specific targeted therapies (Table 4) [155,156].

Currently, conventional cytotoxic agents (such as temozolomide, platinum acetylacetonate, carboplatin, gemcitabine, cytarabine, vincristine and cisplatin), kinase inhibitors (such as erlotinib, ribociclib and other compounds), immunotherapies (including Nivolumab and bevacizumab, among others), and immunomodulators such as CAR T-cells and vaccines are being used alone or in combination with other therapies in various clinical trials to determine their efficacy in the treatment of PBTs and other pediatric CNS tumors (Table 4). Interestingly, there are different clinical trials to identify the appropriate treatment for the different pediatric neurological tumors according to their molecular features; however, most of them were initiated before the establishment of the new classification criteria and maintain the classic nomenclature (pHGG/pLGG and former type descriptions) (Table 4). Indeed, the Pediatric MATCH Screening Trial searches for correlations between drug efficacy and specific mutations in a series of PBTs and other solid tumors (NCT03155620). Another interesting preclinical initiative involves the use of computational tools to reposition already approved drugs targeting PBT cancer stem cells and stemness signaling pathways [157].

Immunotherapy is also being explored for the treatment of cancer [158]. Thus, bevacizumab, which inhibits the VEGFR, has been shown to be effective against several types of cancer, increasing progression-free survival (PFS) in adult patients with glioma but without significantly enhancing median survival [159]. Depatuxizumab mafadotin (ABT-414) is a drug-conjugated antibody that preferentially binds to EGFR-amplified cells (such as GBM cells), and this immunotherapy, alone or in combination with temozolomide, has been under study in pediatric patients with recurrent GBM, but none of the enrolled patients showed a complete or partial response due to tumor progression, without the possibility of completing the treatment (study results published on clinicaltrials.gov: NCT02343406) [160]. Although the efficacy of the antibody therapies has yet to be determined in the treatment of PBTs, different immunomodulators are also under study. Poly-ICLC has been used to treat pLGGs (NCT01188096), either alone or in combination with synthetic peptides such as neoepitope-based personalized vaccines in patients with recurrent brain tumors (NCT03068832) [161]. Moreover, several trials use chimeric antigen receptor (CAR) T cells for PBTs, such as those directed against antigens such as HER2, EGFR806 and B7-H3, whose elevated expression in PBTs supports their potential as therapeutic targets, as acknowledged by the FDA in 2017 [162]. As for immune checkpoint inhibitors, to date no such inhibitors exist for the treatment of PBTs, although promising results were obtained in the first-in-child phase I clinical trial with indoximod plus temozolomide, with improved clinical outcomes in newly diagnosed DIPG patients (median OS and 12-months OS) (NCT02502708, [163]). Indoximod is currently in phase II studies for the treatment of PBTs (Table 4).

Modified viral tools [164] and several PBT cancer vaccines [165,166] are other approaches currently in phase I studies for the treatment of different pediatric neurological tumors, which is a sign of a new trend of innovative therapeutic development.

Lipids have commonly been used as drug delivery vehicles for other molecules [167,168]. However, during recent years, their potential as therapeutic targets has been unveiled by the relevance of lipid metabolism processes in several types of cancer, including lipogenesis, lipolysis, fatty acid oxidation, lipid uptake, and lipid desaturation [169,170]. One example would be CLR131, a radiolabeled molecule that has shown its ability to cross the BBB and provide preliminary activity in children and adolescents with relapsed or refractory cancers, specifically high-grade gliomas (HGGs) and high-risk neuroblastomas, by targeting the preference among malignant cells for phospholipid ethers (NCT03478462). Despite an increasing number of publications pointing to lipid metabolism as a promising therapeutic target in cancer, translation of this potential into actual clinical trials remains scarce. As reviewed by Yan Fu et al. [171], several molecules targeting fatty acid, cholesterol or phospholipid metabolism are under development, mostly at the preclinical stage (Table 5).

Melitherapy is a promising new approach to combat tumors by means of the use of lipids or hydrophobic molecules modulating the composition/structure of lipid membranes as well as lipid metabolism, with potentially high efficacy and safety, suggesting that it could be an appropriate approach to treat PBTs [46,49,170]. Although these are small molecules amenable to production via chemical synthesis or semi-synthetic procedures, their unique mechanisms of action differ from those of more conventional chemotherapeutic agents. The benefits and safety associated with melitherapy recently led to this approach entering the therapeutic arena to treat PBTs and other CNS tumors. Indeed, two different melitherapy agents are currently in phase I clinical studies (LAM561 and BXQ-350) to confirm the safety profile shown in adults and to determine their efficacy in the treatment of the PBTs.

LAM561 (2-hydroxyoleic acid, 2OHOA) has shown clinical activity in adult patients with glioma and other advanced solid tumors and also proved its safety in phase I and II clinical trials, alone and in combination with TMZ, in contrast with most anticancer drugs (clinicaltrials.gov identifier #NCT01792310 and NCT03867123); in addition, its efficacy is being assessed in a phase II/III trial for the treatment of newly diagnosed glioblastoma in adults in combination with the standard of care (radiotherapy and TMZ, clinicaltrials.gov identifier #NCT04250922). The excellent safety profile and promising efficacy of this hydroxylated fatty acid has led to the investigation of its potential use against PBTs in a phase I/II trial (NCT04299191). Significantly, LAM561 was shown to normalize membrane lipid composition, disrupting the association of key peripheral membrane proteins involved in the propagation of messages that drive cancer cell proliferation [190]. LAM561 triggers macroautophagic death of the glioma cells by inducing a relevant reduction in pRb phosphorylation and dihydrofolate reductase expression [190,191], and by dissociating K-Ras from the plasma membrane, all in all inhibiting the MAPK, CDK and PI3K pathways [192].

On the other hand, BXQ-350 (SapC–DOPS) is a nanosome composed of the lysosomal protein saposin C (SapC) and dioleoylphosphatidylserine (DOPS) that targets the surface PS exposed by cancer cells and ceramide-enriched membranes. It is then internalized by endocytosis and induces cell death (necrosis, apoptosis and autophagy) through lysosome activation [193,194]. In addition, the treatment of cancer cells with BXQ-350 sensitizes the cells to irradiation [195]. After showing a good safety profile and promising clinical activity in a phase I clinical trial in adult patients with refractory solid tumors or HGG (NCT02859857), BXQ-350 is currently being investigated to extend these results in a phase I trial in children with newly diagnosed DIPG or diffuse midline glioma (NCT04771897).

Interestingly, valproic acid, a short branched fatty acid that is used as an anticonvulsant, increases the PFS and OS of pediatric DIPG patients [196]. Its hydrophobic nature suggests that, in addition to histone deacetylase inhibition, it may also act through other mechanisms of action related to melitherapy [197]. Furthermore, this study highlights the efficacy of molecules that regulate lipid metabolism alone or in combination with conventional chemotherapy agents, such as temozolomide, for the treatment of brain tumors.

In general, all drugs whose anticancer mechanism of action targets lipid metabolism or is related to cell membrane modulation could be categorized as melitherapy agents. Further examples are farnesyl transferase inhibitors, that prevent the localization of peripheral signaling proteins bearing a farnesyl moiety to the plasma membrane (e.g., Ras).

Ras is a member of the MAPK axis and is regulated by different RTK and growth factor receptors (mutated in different kind of pediatric CNS tumors, see Table 1), as well as by the H3K27M mutation present in the diffuse midline glioma H3 K27-altered, inducing its aberrant activation [198]. Ras localization at the plasma membrane is necessary to propagate growth signals from tyrosine kinase receptors to Raf and other effectors involved in cancer cell proliferation [199]. In this regard, farnesyl transferase inhibitors prevent the localization of peripheral signaling proteins bearing a farnesyl moiety to the plasma membrane, e.g., Ras, and so could act as melitherapeutic drugs. Indeed, the combination of farnesyl transferase inhibitors, tipifarnib and sorafenib, has been studied in adult patients with GBM [200], while lonafarnib has been investigated in children with recurrent or progressive brain tumors (NCT00015899). Despite their limited efficacy, the lack of serious adverse effects suggests that they could be used in combination with other compounds.

Besides the molecules that have been and are already being explored in patients (adult or pediatric) for the treatment of CNS tumors, there are also different compounds that can be endorsed in melitherapy in preclinical research (Table 5). This is the case for ophiobolin A, which has shown promising results against GB in an orthotopic model, by destabilizing the membrane after covalent modification of phosphatidylethanolamine [201].

Many of these molecules directly affect lipid metabolism or biosynthesis, such as fluoxetine, which inhibits sphingomyelin phosphodiesterase 1 (SMPD1) in GBM cells and prevents the conversion of sphingomyelin to ceramide [179] or YTX-7739 and CAY10566, which act as stearoyl CoA desaturase (SCD) inhibitors and trigger lipotoxicity, impairing de novo lipid synthesis [184]. A combination of GSK126 and atorvastatin, a cholesterol biosynthesis inhibitor, showed good results in a murine DIPG model [173], and LXR-623, an agonist of LXR, has been found to be very effective in killing GBM cells in a xenograft model by depleting cholesterol levels [188]. Fatty acid uptake by pediatric ependymoma cells in a 3D spheroid has been also targeted using GW9662 through inhibiting the brain-lipid-binding protein (BLBP or FABP7) gene expression [176]. In addition to molecules targeting lipid-related pathways, other drugs of a lipid nature are being investigated. Ljungblad et al. showed that omega-3 fatty acids can suppress the growth of tumors in pediatric medulloblastoma cells by altering fatty acid composition and decreasing CRYAB expression levels [177]. In addition, a triple-front approach using LAU-0901 (a PAF agonist), avastin (VEGF suppressor) and elovanoids (synthetic dihydroxylated derivatives of PUFAs acting as lipid mediators) has demonstrated therapeutic efficacy suppressing GBM tumors in orthotopic xenograft mice models [181].

Lipid-metabolism-based therapies may have the advantage over single-protein-based therapies in that there are common lipidomic profiles for several tumor types that promote tumor growth, as described above. However, some of the mutated genes are specific to certain types of diseases and therefore targeting metabolic energy sources or the important energy dependence of PBT may represent an opportunity for therapeutic intervention in these conditions. Furthermore, lipids not only represent a source of energy, but also define the membrane structure and its recruitment ability for peripheral signaling proteins, which can activate or inhibit cancer cell proliferation. Thus, monotherapy or combinational therapy with agents targeting lipid metabolic pathways with antiproliferative drugs could be more efficacious against glioma and may show efficacy against PBTs. Moreover, targeting lipid metabolism-based tumor growth could in turn revert the neoplastic proliferative phenotype, while targeting oncogenes could have an impact on the lipid metabolism reprogramming.

## 6. Concluding Remarks

Currently, therapies to combat adult cancers are also used against pediatric neurological tumors, yet the standard of care for each type of tumor may differ depending on the molecular characteristics and location of the tumor as well as the age of the patient. The latest data concerning the molecular similarities and differences found between child and adult tumors will have important implications for the treatment of PBTs. Beyond toxic chemotherapeutic compounds, radiotherapy and surgery, innovative approaches based on the molecular signature of pediatric cancers may lead to new, safer and more efficacious therapies. Moreover, lipidic molecules, enzymes related to lipid metabolism, and the lipid composition of tumors represent potential biomarkers for the prognosis and characterization of pediatric CNS tumors, as well as therapeutic targets for a new class of compounds with high safety and potential efficacy in their treatment.

## Figures and Tables

**Table 1:** Molecular alterations in pediatric CNS tumors and their association with the lipid metabolism and composition.

Type of Tumor	WHO Grade	Main Molecular Alterations	Codified Proteins Affected by Lipid Metabolism or Lipid Composition Regulation	Percentage of Cases
GLIOMAS, GLIONEURONAL TUMORS AND NEURONAL TUMORS (excluding adult-type diffuse glioma)				
Pediatric-type diffuse high-grade glioma				11.1
Diffuse midline glioma H3 K27-altered	3	H3 K27, *TP53*, *ACVR1*, *PDGFRA*, *EGFR*, *EZHIP*	P53—regulator of lipid metabolism in cancer [11]. Mutations on TP53 provide lipolytic activity to P53 [12]. EGFR is regulated by palmytoilation at Cys1049 and Cys1146 [13].	
Diffuse hemispheric glioma, H3 G34-mutant	4	H3 G34, *TP53*, *ATRX*	P53—regulator of lipid metabolism in cancer [11]. Mutations on TP53 provide lipolytic activity to P53 [12]. Atrx—transcriptional factor targeting lipid metabolism mediators.	
Diffuse High-grade glioma H3-wild-type and IDH-wild-type	4	IDH-wildtype, H3-wildtype, *PDGFRA*, *MYCN*, *EGFR* (methylome)	IDH-wild type—IDH1 activity is critical for lipid biosynthesis and its inactivation compromises tumor growth [14] MCYN—Lipid desaturation-associated endoplasmic reticulum stress regulates MYCN gene expression [15]. EGFR is regulated by palmytoilation at Cys1049 and Cys1146 [13].	
Infant-type Hemispheric glioma	4	NTRK family, *ALK*, *ROS*, *MET*	NTRK, Alk, Ros and MET are transmembrane proteins.	
Pediatric-type diffuse Low-grade gliomas				25–30
Diffuse astrocytoma, MYB or MYBL1 altered	1–2	*MYB*, *MYBL1*		
Angiocentric glioma	1	*MYB*, BRAF V600E mut	BRAF V600E mut—induction of lipid droplet accumulation [16]	
Polymorphous low-grade neuroepithelial tumor of the young	1	*BRAF*, FGFR family	BRAF—the lipogenic pathway is a key mediator of oncogenic BRAF. Inhibition of oncogenic BRAF caused an increase in the proportion of poly-unsaturated membrane phospholipid species at the expense of saturated and mono-unsaturated phospholipids [17]. FGFR—transmembrane protein	
Diffuse low-grade glioma MAPK pathway-altered	1	*FGFR1*, *BRAF*	BRAF—the lipogenic pathway is a key mediator of oncogenic BRAF. Inhibition of oncogenic BRAF caused an increase in the proportion of poly-unsaturated membrane phospholipid species at the expense of saturated and mono-unsaturated phospholipids [17]. FGFR1—transmembrane protein. The altered lipid structure allows one to factor in the protein–lipid interactions and the biophysical properties of the resulting membranes into the regulation of signal transduction pathways such as the MAPK pathway [18].	
Circumscribed astrocytic gliomas				17.6
Pilocytic astrocytoma	1	*KIAA1549-BRAF*, *BRAF*, *NF1*	BRAF—the lipogenic pathway is a key mediator of oncogenic BRAF. Inhibition of oncogenic BRAF caused an increase in the proportion of poly-unsaturated membrane phospholipid species at the expense of saturated and mono-unsaturated phospholipids [17]. NF1—phospholipid binding protein [19].	
High-grade astrocytoma with piloid features	3–4	*BRAF*, *NF1*, *ATRX*, *CDKN2A/B* (methylome)	BRAF—the lipogenic pathway is a key mediator of oncogenic BRAF. Inhibition of oncogenic BRAF caused an increase in the proportion of poly-unsaturated membrane phospholipid species at the expense of saturated and mono-unsaturated phospholipids [17]. NF1—phospholipid binding protein [19]. Atrx—transcriptional factor targeting lipid metabolism mediators.	
Pleomorphic xanthoastrocytoma	2	*BRAF*, *CDKN2A/B*	BRAF—the lipogenic pathway is a key mediator of oncogenic BRAF. Inhibition of oncogenic BRAF caused an increase in the proportion of poly-unsaturated membrane phospholipid species at the expense of saturated and mono-unsaturated phospholipids [17].	
Subependymal giant cell astrocytomas (SEGA)	1	*TSC1*, *TSC2*	TSC1—inhibition of lipophagy or its downstream catabolic pathway reverses defective phenotypes caused by Tsc1-null NSCs and reduces tumorigenesis in mouse models [20]. TSC2—TSC2-deficient cells have enhanced choline phospholipid metabolism [21]	
Astroblastoma, MN1-altered	3–4	*MN1*		
Ependymal tumors				10
Subependymoma	1–2			
Supratentorial ependymomas ZFTA fusion-positive	2	*ZFTA*, *RELA*		
Supratentorial ependymomas, YAP1 fusion positive	2–3	*YAP1*, *MAML2*	YAP1 positively regulates numerous genes related to cancer stemness and lipid metabolism [22]	
Posterior fossa ependymomas, group PFA (EZHIP mutation)	2–3	H3 K27me3, *EZHIP* (methylome)		
Posterior fossa ependymomas, group PFB	2			
Spinal ependymomas, MYCN-amplified	3	*NF2*, *MYCN*	NF2—lipid binding results in the open conformation of neurofibromin 2 [23] MYCN—lipid desaturation-associated endoplasmic reticulum stress regulates MYCN gene expression [15].	
Myxopapillary ependymoma	2			
Neuronal and glioneuronal tumors				4.4
Dysembryoplastic neuroepithelial tumors (DNET)	2	*FGFR1*	FGFR1—transmembrane protein	
Gangliogliomas	1–2			
Diffuse glioneuronal tumor with oligodendroglioma-like features and nuclear clusters (DGONC)	2	*Chromosome 14, (methylome)*		
Myxoid glioneuronal tumor (MGT)	2	*PDFGRA*		
Multinodular and vacuolating tumor (MVNT)	1	MAPK pathway	An altered lipid structure allows one to factor in the protein–lipid interactions and the biophysical properties of the resulting membranes into the regulation of signal transduction pathways such as the MAPK pathway [18]	
Rosette-forming glioneuronal tumor	1	*FGFR1*, *PIK3CA*, *NF1*	FGFR1—transmembrane protein. PIK3CA—phospholipid binding protein. NF1—phospholipid binding protein [19].	
Myxoid glioneuronal tumor	1	*PDFGRA*		
Diffuse leptomeningeal glioneuronal tumor	1–3	*KIAA1549-BRAF* fusion, 1p (methylome)	BRAF—the lipogenic pathway is a key mediator of oncogenic BRAF. Inhibition of oncogenic BRAF caused an increase in the proportion of poly-unsaturated membrane phospholipid species at the expense of saturated and mono-unsaturated phospholipids [17].	
Gangliocytoma	1			
Dysplastic cerebellar gangliocytoma (Lhermitte-Duclos disease)	1	*PTEN*	PTEN—phospholipid binding protein which also interacts with FABP4 [24]	
Central neurocytoma	2			
Extraventricular neurocytoma	2	FGFR (*FGFR1-TACC1* fusion), IDH-wild type	FGFR—transmembrane protein	
Cerebellar liponeurocytoma	2			
CNS EMBRYONAL TUMORS				
Medulloblastoma				20.0
Medulloblastoma, molecularly defined	4			
Medulloblastoma, WNT-activated	4	*CTNNB1*, *APC*	CTNNB1—ß-catenin strongly promotes ß-oxidation [25]	
Medulloblastoma, SHH-activated and TP53-wild-type	4	*PTCH1*, *SUFU*, *SMO*, *MYCN*, *GLI2* (methylome)	PTCH1, GLI2—lipid metabolism has a profound influence on both hedgehog signal transduction and the properties of the ligands themselves [26] SMO—Hh signaling transduces to SMO through modulating its cholesterylation [27].	
Medulloblastoma, SHH-activated and TP53-mutant	4	*TP53*, *PTCH1*, *SUFU*, *SMO*, *MYCN*, *GLI2* (methylome)	TP53—mutations on TP53 provide lipolytic activity to P53 [12]. PTCH1, GLI2—Lipid metabolism has a profound influence on both hedgehog signal transduction and the properties of the ligands themselves [26] SMO—Hh signaling transduces to SMO through modulating its cholesterylation [27].	
Medulloblastoma, non-WNT/non-SHH	3–4	*MYC*, *MYCN*, *PRDM6*, *KDM6A* (methylome)	MYC—fatty acids are inhibitors of the DNA binding of c-Myc/Max dimer [28] MCYN—lipid desaturation-associated endoplasmic reticulum stress regulates MYCN gene expression [15,29].	
Medulloblastoma, histologically defined	3–4			
Other CNS embryonal tumors				
Atypical teratoid/rhabdoid tumor (ATRT)	4	*SMARCB1*, *SMARCA4*	SMARCB1—also known as SWI/SNF-related matrix-associated protein, related also to SMARCA4—BAF60a and BAF60c, two subunits of the SWI/SNF chromatin-remodeling complexes, are important for maintaining hepatic lipid metabolism. SWI/SNF complex might be targeted to develop drugs aimed at regulation of lipid homeostasis in hepatic steatosis [30].	
Cribriform neuroepithelial tumor (provisional type)	3–4			
Embryonal Tumor with Multilayer Rosettes (ETMR)	4	C19MC, *DICER1*	DICER1—the loss of miRNAs resulting from Dicer1 deficiency greatly contributes to the progression of many diseases, including lipid dysregulation [31].	
Neuroblastoma, FOXR2-activated	4	*FOXR2*		
CNS tumor with BCOR internal tandem duplication	4	*BCOR*		
Embryonal tumor NEC/NOS	4			
TUMORS OF THE SELLAR REGION				
Craniopharyngioma				4.0
Adamantinomatous craniopharyngioma	1	*CTNNB1*	CTNNB1—ß-catenin strongly promotes ß-oxidation [25]	
Papillary craniopharyngioma	1	*BRAF*	BRAF—the lipogenic pathway is a key mediator of oncogenic BRAF. Inhibition of oncogenic BRAF caused an increase in the proportion of poly-unsaturated membrane phospholipid species at the expense of saturated and mono-unsaturated phospholipids [17].	
Pituitary endocrine tumors	3.9			
Pituitary blastoma	1–4	*DICER1*	Dicer—Dicer disruption caused a marked decrease in microsomal triglyceride transfer protein, long-chain fatty acyl-CoA ligase 5, fatty acid binding protein, and very-long-chain fatty acyl-CoA dehydrogenase [32].	
MELANOCYTIC TUMORS				
Meningeal melanocytosis and melanomatosis	1–3			2.5
GERM CELL TUMORS				
	1			3.7
MENINGIOMAS				
Meningioma	1–3	*NF2, AKT1, TRAF7, SMO, PIK3CA; KLF4, SMARCE1,* *BAP1 in subtypes; H3K27me3; TERT promoter, CDKN2A/B in CNS WHO grade 3*	NF2—lipid binding results in the open conformation of neurofibromin 2 [23]. SMO—Hh signaling transduces to SMO through modulating its cholesterylation [27]. PIK3CA—phospholipid binding protein. KLF4—regulates cholesterol metabolism by endothelial cells [33].	2.9
CHOROID PLEXUS TUMORS				
				2.3
Plexus papilloma	1			
Atypical plexus papilloma	2			
Plexus carcinoma	3			
Plexus papilloma	1			
PINEAL TUMORS				
				3–11
Pineocytoma	1			
Pineoblastoma	4			
Papillary tumor of pineal region	2–3			
OTHER/UNCLASSIFIED TUMORS				
				4.9

**Table 2:** Genes involved in lipid metabolism referred to herein.

Official Symbol	Enzyme Name
ACER1	ASAH1; Alkaline Ceramidase 1
ACER3	ASAH3; Alkaline Ceramidase 3
ACSL1	Acyl-CoA Synthetase Long-Chain Family Member 1
ACSL3	Acyl-coA synthetase Long Chain Family member 3
ACSL4	Acyl-coA synthetase Long Chain Family member 4
ACSL5	Acyl-coA synthetase Long Chain Family member 5
AHR	Aryl Hydrocarbon Receptor
ALDH3A2	Fatty Aldehyde dehydrogenase
ASAH2	Ceramidase, non-lysosomal
CD36	CD36 Molecule
CEPT1	Choline/Ethanolamine Phosphotransferase 1
CERS1	LASS1, Ceramide Synthase 1
DEGS1	Delta 4-Desaturase, Sphingolipid 1
FA2H	Fatty Acid 2-hydroxylase
FABP5	Fatty Acid Binding Protein 5
FABP7	Fatty Acid Binding Protein 7
FADS1	Fatty Acid Desaturase 1
FADS2	Fatty Acid Desaturase 2
FASN	Fatty Acid synthase
FFAR1	Free Fatty Acid Receptor 1; GPR40
FFAR2	Free Fatty Acid Receptor 2; GPR43
FFAR3	Free Fatty Acid Receptor 3; GPR41
FFAR4	Free Fatty Acid Receptor 4; GPR120; O3FAR1
GALC	Galactosylceramidase
GPR42	G Protein-Coupled Receptor 42 (Gene/Pseudogene); FFAR1L
HACL1	2-hydroxypythanoyl-coA-lyase, 2-hydroxyacyl-CoA lyase 1
HSPA5	BiP, GRP78; Heat Shock Protein Family A (Hsp70) Member 5
LPAR1	LPA1; Lysophosphatidic Acid Receptor 1
LPAR2	LPA2; Lysophosphatidic Acid Receptor 2
LPAR3	LPA3; Lysophosphatidic Acid Receptor 3
LPAR4	LPA4; Lysophosphatidic Acid Receptor 4
LPAR5	LPA1; Lysophosphatidic Acid Receptor 5
LPAR6	LPA1; Lysophosphatidic Acid Receptor 6
NR1H3	LXRA; Liver X Nuclear Receptor Alpha Variant 1
NSMAF	N-Smase; Neutral Sphingomyelinase Activation Associated Factor
PEMT	Phosphatidylethanolamine N-Methyltransferase
PHYH	Phytanoyl-CoA 2-hydroxylase
PPARa	Peroxisome Proliferator Activated Receptor Alpha
PPARb	Peroxisome Proliferator Activated Receptor Beta
PPARd	Peroxisome Proliferator Activated Receptor Delta
PPARg	Peroxisome Proliferator Activated Receptor Gamma
S1P1	Sphingosine-1-Phosphate Receptor 1
S1P2	Sphingosine-1-Phosphate Receptor 2
S1P3	Sphingosine-1-Phosphate Receptor 3
S1P4	Sphingosine-1-Phosphate Receptor 4
S1P5	Sphingosine-1-Phosphate Receptor 5
SAMD8	SMSr; CEP Synthase; Sterile Alpha Motif Domain Containing 8
SCD	Stearoyl CoA desaturase
SMS1	Sphingomyelin synthase 1
SMS2	Sphingomyelin synthase 2
SGPL1	Sphingosine-1-Phosphate Lyase 1
SMPD1	Acid Sphingomyelinase
SMPD2	Neutral sphingomyelinase 1
SMPD3	Neutral sphingomyelinase 2
SMPD4	Neutral sphingomyelinase 3
SMPDL3A	Acid Sphingomyelinase-Like Phosphodiesterase 3a
SMPDL3B	Acid Sphingomyelinase-Like Phosphodiesterase 3b+C1:C63
SPHK1	Sphingosine kinase 1
SPHK2	Sphingosine kinase 2
SPTLC3	Serine palmitoyl Transferase, long chain subunit 3
TLR2	Toll-Like Receptor 2
UGCG	UDP-Glucose Cer Glucosyltransferase (GluCer synthase)

Genes that were not present in the array from the database used are indicated in red.

**Table 3:** Cases of transcription factors controlling lipid metabolism mediator expression.

Official Symbol	Transcription Factor Name	Target Lipid Metabolism Mediator
AHR	Aryl Hydrocarbon Receptor	To be determined [94]
AP-1	Activator protein 1	ASAH2 [95] SPHK1 [96]
AP-2	Transcription Factor AP-2 Alpha	ASAH2 [95]
Atrx	Alpha Thalassemia/Mental Retardation Syndrome X-Linked	Several complexes along the chromosome maintain different states of chromatin [97]
Atf-4	Activating Transcription Factor 4	SPHK2 [98,99]
BCL11B	B-Cell Lymphoma/Leukaemia 11B	SMPD2 [100]
CREB	CAMP Responsive Element Binding Protein 1	SPHK2 [98]
E2F	E2F Transcription Factor 1	SPHK1 [101]
Fos	FBJ Murine Osteosarcoma Viral Oncogene Homolog	SMPD3 [102,103,104,105]
GATA	GATA Transcription Factor	ASAH2 [95]
Hey1	Hes Related Family BHLH Transcription Factor With YRPW Motif 1	ACVR1 [106]
HIF1α	Hypoxia-inducible factor 1-alpha	PDGFRA [107]
HIF2α	Hypoxia-inducible factor 2-alpha	SPHK1 [108]
IRF1	Interferon-regulatory factor-1	ASCL4 [109,110]
LMO2	LIM domain only 2 rhombotin-like 1	SPHK1 [111]
NF-Y	Nuclear factor Y	ASAH2 [95] FASN [112,113]
Oct-1	POU Class 2 Homeobox 1	ASAH2 [95]
SP1	Specificity Protein 1	ASAH2 [95] UGCG [95,114]
ZBTB7A/LRF	Zinc Finger And BTB Domain Containing 7A/Lymphoma Related Factor	ACVR1 [106]

**Table 4:** Selection of current clinical trials in pediatric patients with brain tumors.

Drug Type	Example Agents	Target	Disease	Pediatric Clinical Trial
Immunomodulators	APX005M	CD40 agonist	GBM, A, CNST, E, DIPG, MB	NCT03389802
	Pomalidomide	TNFa	CNSTS	NCT03257631
	Indoximod	IDO, mTOR	E, MB, GBM, DIPG	NCT05106296 NCT04049669
	NKTR-214	CD122 IL2 pathway agonist	E, HGG, MB, PBTs	NCT04730349
Antibodies	Magrolimab	CD47	PBTs	NCT05169944
	Avelumab	PD-L1	CNSTs	NCT05081180
	Nivolumab	PD-1 receptor	CNSTs	NCT03838042 NCT04500548
	Ipilimumab	CTLA-4	CNSTs	NCT04500548
	Bevacizumab	VEGF-A	PBTs	NCT02698254
CAR T Cells and other cellular immunotherapies	HER2-specific CAR T cell locoregional Immunotherapy	HER2	G, E, MB, GCT, ATRT, PB	NCT03500991
	EGFR806-specific CAR T cell locoregional Immunotherapy	EGFR	G, E, MB, GCT, ATRT, PNET, CPC, PB	NCT03638167
	B7-H3-specific CAR T Cell locoregional Immunotherapy	B7H3	DIPG, DMG, E, MB, GCT, ATRT, CPC, PB, G	NCT04185038
	GD2-CART01 (iC9-GD2-CAR T-cells)	Disialoganglioside GD2	MB, PBTs	NCT05298995
	IL13Ralpha2-specific hinge-optimized 41BB-co-stimulatory CAR truncated CD19	IL13Ralpha2	PBTs	NCT04510051
	Haploidentical transplant and donor NK cell infusion		CNSTs	NCT02100891
	Bone marrow-derived allogenic mesenchymal stem cells infected with an oncolytic adenovirus, ICOVIR-5	pRB pathway	DIPG, MB	NCT04758533
Vaccines	PEP-CMV	CMV antigen	HGG, DIPG, MB	NCT03299309 NCT05096481
	Personalized neoantigen DNA vaccine		DMG, DIPG	NCT03988283
	rHSC-DIPGVax (neo-antigen heat schock protein vaccine)		DMG, DIPG	NCT04943848
	Dendritic cell vaccination: WT1 mRNA-loaded autologous monocyte-derived DCs		HGG DIPG	NCT04911621
	Immunomodulatory DC vaccine		DIPG, GBM	NCT03914768
	SurVaxM	Survivin	MB, GBM, AA, A, NOS, AO, AE, E, DIPG	NCT04978727
	K27M peptide		DIPG, DMG	NCT02960230
Viral Therapy	HSV G207 oncolytic herpes simplex virus-1 (HSV)		CNSTs	NCT03911388 NCT02457845
	Wild-type reovirus (reolysin)		HGGs	NCT02444546
	Polio/rhinovirus recombinant (PVSRIPO)	CD155 nectin-like molecule-5	CNSTs	NCT03043391
	DNX-2401 oncolytic adenovirus	Integrins	BSG, DIPG	NCT03178032
Conventional chemotherapeutics	Mebendazole:	Tubulin	MB, A, GB, AA, Brain Stem Neoplasms, O, AO, G	NCT02644291
	PTC596	Tubulin	DIPG, HGG	NCT03605550
Antimetabolites	Pemetrexed	Folate analog	MB	NCT01878617
	Hydroxyurea	RRM2	G, GBM	NCT03463733
New chemotherapeutics	Marizomib	Proteasome	DIPG, BSG, PBTs	NCT04341311
	ALRN-6924	MDM2/MDMX	PBTs	NCT03654716
	Curaxin CBL0137	FACT	DMG, DIPG, CNSTs	NCT04870944
Kinase Inhibitors	CX-4945 silmitasertib	CK2	MB	NCT03904862
	Prexasertib	Chk1	MB	NCT04023669
	9-ING-41	GSK 3β	PBTs	NCT04239092
	Trametinib	MEK1, MEK2	PBTs	NCT03434262
	Ibrutinib	Bruton’s tyrosine Kinase	E, MB, GBM	NCT05106296
	Lenvatinib	VEGFR1, 2 and 3, FGFR1, 2, 3 and 4, PDGFR alpha, c-Kit, RET proto-oncogene	CNSTs	NCT05081180 NCT03245151
	Alectinib	ALK	CNSTs	NCT04774718
	Larotrectinib	Tropomyosin receptor kinases	CNSTs	NCT03213704 NCT03834961 NCT03155620
	Repotrectinib (TPX-0005)	ALK, ROS	CNSTs	NCT04094610
Downstream signaling pathway inhibitors	Vemurafenib	B-Raf. BRAFV600	G	NCT01748149 NCT03220035
	Entrectinib	TRKA, TRKB, TRKC, ROS1, ALK	CNSTs	NCT02650401
	ONC206	Stress response, DRD2/ClpP	DMG, CNSTs	NCT04732065
	Everolimus immunosupr	mTor, FKBP-12	HGG, PNET	NCT03245151
	Sirolimus immunosupr	mTor, FKBP-12	CNSTs	NCT02574728
	GDC-0084	PI3K/mTor	CNSTs	NCT03696355
	WP1066	JAK/STAT3	PBTs	NCT04334863
	Indoximod	IDO, mTOR	E, MB, GBM	NCT05106296
	MEK162	Ras/Raf/MEK	LGG	NCT02285439
	Trametinib	MEK1/2	PBTs	NCT04485559 NCT03363217 NCT05180825 NCT02684058 NCT04201457
Developmental pathway inhibitors	Vismodegib	SMO	MB	NCT01878617
Cell Death Pathway inducers	ONC201	TRAIL, ISR	DIPG, DMG, HGG	NCT05009992 NCT05580562
Angiogenesis inhibitor	Recombinant human endostatin (rh-ES)	Ras, Raf, VEGF, VEGFR2	LGG	NCT04659421
Epigenetic therapy	BMS-986158 and BMS-986378	Bromodomain (BRD) and extra-terminal domain (BET)	PBTs	NCT03936465
	RRx-001	DNMT and global methylation	PBTs	NCT04525014
	Panobinostat	HDAC	DIPG, BSG, PBTs	NCT02717455 NCT04341311
			MRT/ATRT	NCT04897880
	Entinostat	Class I and IV HDAC	CNSTs	NCT03838042
	Tazemetostat	EZH2	CNSTs	NCT03213665
	Vorinostat	HDAC	BSG, A, CAA, CSCN	NCT01236560
	BMS-986158	Bromodomain and extra-terminal (BET) proteins	PBTs	NCT03936465
Melitherapy	2-hydroxyoleic acid	Plasma membrane composition	PBTs	NCT04299191
	BXQ-350	Plasma membrane sphingolipid modulation	DIPG, DMG PBTs	NCT04771897 NCT04404569
Radiolabeled drugs	Radiolabeled phospholipid drug conjugate: CLR 131 radioiodinated phospholipid ethers (PLEs)	Lipid rafts of cancer cell membranes	PBTs,	NCT03478462
	Peptide receptor radionuclide: lutathera (177Lu-DOTATATE)	Somatostatin receptors	CNSTs	NCT05278208
	Radiolabelled monoclonal antibody: iodine I 131 MOAB 8H9	4Ig-B7-H3	CNSTs	NCT00089245

Abbreviations: A: astrocytoma, AA: anaplastic astrocytoma, AE: anaplastic ependymoma, ATRT: atypical teratoid rhabdoid tumor, AO: anaplastic oligodendroglioma, BSG: brain stem glioma, CAA: cerebellar anaplastic astrocytoma, Chk1: checkpoint kinase 1, CK2: protein casein kinase II, ClpP: human mitochondrial caseinolytic protease P, CNSTs: central nervous system tumors, CPC: choroid plexus carcinoma, CSCN: childhood spinal cord neoplasm, DIPG: diffuse intrinsic pontine glioma, DMG: diffuse midline gliomas, DNMT: DNA methyltransferase, DRD2: dopamine receptor D2, E: ependymoma, G: glioma, FACT: facilitates chromatin transcription complex, G: glioma, GBM: glioblastoma multiforme, GCT: germ cell tumor, HDAC: histone deacetylase, HGG: high-grade glioma, IDO: indoleamine 2,3-dioxygenase, ISR: TRAIL, integrated stress response LGG: low-grade glioma, MB: medulloblastoma, MRT: malignant rhabdoid tumor, O: oligodendroglioma, PB: pineoblastoma, PBTs: pediatric brain tumors, PD-L1: programmed death-ligand 1, pRB: retinoblastoma tumor suppressor protein, RRM2: M2 protein subunit of ribonucleotide reductase.

**Table 5:** Selection of pre-clinical stage therapeutic drugs of lipid nature (Italic) or targeting lipid-related pathways in brain tumors.

Category	Drug Agent	Family	Target	Affected Pathways	Disease	Model	Reference
Pediatric	Cordycepin	Nucleoside derivative	miR-33	Lipid metabolism	MB	Orthotropic xenograft	[172]
	GSK126+ Atorvastatin	Small molecule inhibitors	EZH2	Cholesterol synthesis	DIPG	Murine orthotopic model	[173]
	ABC294640	Small molecule inhibitor	SphK2	Sphingolipid metabolism	DIPG	SF8628 and SF7761 Soft agar	[174]
	Carbenoxolone + palbociclib	Small molecule inhibitors	HSD11β2- CDK4/6	Oxysterol biosynthesis	MB	Transgenic	[175]
	GW9662	Small molecule agonist	BLBP	Fatty acid uptake	E	3D spheroid	[176]
	*ω3-LCPUFA*	Fatty acids	CRYAB	Protein folding	MB	Xenograft	[177]
	*Erucylphosphocholine*	Ether lipid	Membrane	Apoptosis	MB	D283 Med	[178]
General	Fluoxetine	Small molecule inhibitor	SMDP-1	Sphingolipid metabolism	GBM	Orthotropic xenograft	[179]
	Triacsin C + Etoximir	Small molecule inhibitors	ACSL1- ACSL3-CPT1	Lipid biosynthesis and fatty acid oxidation	Mesenchymal GBM	Xenograft	[180]
	LAU-0901 + Avastin + *Elovanoids*	Small molecule + synthetic lipids	PAFR	Tumor cell proliferation	GBM	Orthotropic xenograft	[181]
	Arachidonyl trifluoromethyl ketone	Small molecule inhibitor	PTRF(cavin-1)	Phospholipid metabolism	GBM	Intracranial Patient-Derived Xenograft Model	[182]
	CAY10566	Small molecule inhibitor	SCD1	Lipogenesis	GBM	Xenograft	[183]
	YTX-7739	Small molecule inhibitor	SCD	Lipogenesis	GBM	Orthotropic xenograft	[184]
	Etomoxir	Small molecule inhibitor	CPT1	Fatty acid oxidation	GBM	Syngeneic	[185]
	Azathioprine	Purine analogue	EGFR-AKT	Lipid metabolism	GBM	Orthotropic xenograft	[186]
	Ophiobolin A	Terpenoid antagonist	PE	Membrane Destabilization	GBM	orthotopic U251-LUC xenograft	[187]
	LXR-623	Small molecule agonist	LXR	Cholesterol metabolism	GBM	Orthotropic xenograft	[188]
	*GM3*	Ganglioside	VEGF	Tumor angiogenesis.	A	CT-2A Matrigel	[189]

Abbreviations: A: astrocytoma, ACSL: adipose acyl-CoA synthetase, AKT: protein kinase B, BLBP: brain lipid-binding protein, CDK: cyclin-dependent kinase, CPT1: carnitine palmitoyltransferase I, CRYAB: alpha-crystallin B chain, DIPG: diffuse intrinsic pontine glioma, E: ependymoma, EGFR: epidermal growth factor receptor, EZH2: enhancer of zeste homolog 2, GBM: glioblastoma, HSD11b2: 11β-hydroxysteroid dehydrogenase, type 2, LXR: Liver X receptor, MB: medulloblastoma, PE: phosphatidylethanolamine, PTRF: polymerase I and transcript release factor, SCD1: stearoyl-CoA desaturase-1, SMDP1: surfactant metabolism dysfunction, pulmonary, type 1, SPHK2: sphingosine kinase 2, VEGF: vascular endothelial growth factor.

## Data Availability

Raw dataset from XENA website is publicily available in the website under the number E-TABM-1107 and was analyzed using the Pedriatic_review_v7.Rmd code file available in the supplementary material of this article.

## Supplementary Materials

The following supporting information can be downloaded at: https://www.mdpi.com/article/10.3390/biomedicines11051365/s1.

## Abbreviations

2OHOA	2-hydroxyoleic acid
ATRT	atypical teratoid/rhabdoid tumor
ATRX	α thalassemia/intellectual disability syndrome X–linked gene
BBB	blood–brain barrier
BRAF	B-Raf proto-oncogene serine/threonineprotein kinase
CAR	chimeric antigen receptor
CNS	central nervous system
CSF	cerebrospinal fluid
DIPG	diffuse intrinsic pontine glioma
DNETs	dysembryoplastic neuroepithelial tumors
ETMR	embryonal tumor with multilayer rosettes
FABP	fatty-acid-binding protein
GBMs	glioblastomas
HGG	high-grade glioma
LGG	low-grade glioma
MAPK	mitogen-activated protein kinase
MRSI	proton magnetic resonance spectroscopic imaging
MS	mass spectroscopy
NMR	nuclear magnetic resonance
OS	overall survival
PA	pilocytic astrocytoma
PBTs	pediatric brain tumors
PFS	progression-free survival
pLGG/HGG	pediatric low-/high-grade glioma
PXA	pleomorphic xanthoastrocytoma
SCD	stearoyl CoA desaturase
SEGA	subependymal giant cell astrocytomas
SHH	Sonic Hedgehog
SMO	smoothened: frizzled class receptor
SM	sphingomyelin
SUFU	suppressor of fused homolog (Drosophila)
TP53	tumor protein p53
WHO	World Health Organization
WT	wild-type
